# Comment on Värendh et al. A Cost-Effectiveness Analysis of the Sentio Bone Conduction Hearing Implant System in the Australian Healthcare Setting. *J. Mark. Access Health Policy* 2026, *14*, 8

**DOI:** 10.3390/jmahp14040059

**Published:** 2026-09-30

**Authors:** Matthias Brunner

**Affiliations:** Cochlear Limited, Macquarie University, Sydney, NSW 2109, Australia; mbrunner@cochlear.com

We read with interest the cost-effectiveness analysis by Värendh et al. [1] comparing the Sentio and Osia bone conduction hearing implant (BCHI) systems in the Australian healthcare setting. While we appreciate efforts to evaluate the recently introduced Sentio system, substantial methodological flaws and unvalidated assumptions, including inconsistency with Sentio’s instructions for use, systematically bias the results in favour of Sentio, undermining the authors’ conclusion of economic dominance over Osia. We highlight four critical concerns that compromise the study’s validity for clinical and health technology assessment (HTA) decision-making.

## 1. Disparate Clinical Evidence Standards

A fundamental flaw is the profound disparity in clinical evidence quality between the two devices. The authors acknowledge that “Sentio is a recently introduced device” with “limited long-term data” yet proceed as though evidence bases were equivalent. This violates HTA principles requiring comparable evidence quality across comparators.

For complication rates—a key driver of both quality-adjusted life years (QALYs) and costs—Osia evidence derives from a systematic review and meta-regression by Caversaccio et al. [2] synthesising 12 studies with patients implanted with an Osia system. In contrast, Sentio evidence relies primarily on a single 51-patient study by Hol et al. [3] with six months follow-up, supplemented by proxy data from predecessor devices.

Critically, the Hol et al. study reported zero explantations at six months [3]. This does not constitute evidence of zero risk but reflects insufficient statistical power to detect rare events in a small sample over a short duration. The authors could have avoided this asymmetry by using the comparable Osia study by Briggs et al. [4] with six-month follow-up, which also showed zero explantations. Instead, they applied disparate evidence standards that inherently favour Sentio.

## 2. Unsupported Equal Utility Assumption

The authors assume identical patient utility (0.76 for the On-BCHI state) for both devices despite having no Sentio-specific utility data. While health-related quality of life utilities were collected in key Osia clinical studies using the Health Utilities Index Mark 3 (HUI-3) instrument, no such data exist for Sentio patients. The equal utility assumption rests entirely on “clinical expertise validation” involving four unidentified international experts through an undisclosed “semi-structured” process.

This validation approach lacks transparency regarding expert identities, potential conflicts of interest, questions posed, evidence reviewed, access to actual Sentio patient-reported outcomes, or validation criteria used. From a clinical and HTA perspective, assuming equal effectiveness without supporting data contradicts evidence-based decision-making principles.

More importantly, this assumption fundamentally undermines the dominance claim. The authors explicitly acknowledge that “incremental QALY differences in the model are driven by modelled event pathways and cost offsets rather than demonstrated differences in clinical effectiveness”. The claimed QALY difference over 15 years (approximately 0.007 QALYs annually, equivalent to 2.7 days of perfect health per year) derives entirely from differences in modelled complication rates, not from superior patient-reported outcomes, better hearing performance, or improved quality of life. Sentio demonstrates no quality of life advantage over Osia according to the model’s own logic.

## 3. Inappropriate Handling of Zero-Event Data

A fundamental statistical flaw concerns the zero-event explantation rates for Sentio. The model assumes zero probability of explantation based on observing no events in 51 patients followed for only six months. In clinical research, when zero events occur in small samples, absence of evidence is not evidence of absence.

This assumption is particularly problematic given its crucial role in driving the QALY advantage. Explantation events transition patients permanently to the Off-BCHI health state with lower quality of life equivalent to pre-operative, unaided hearing. Since the authors assume equal utility with either functioning device, the QALY benefit attributed to Sentio stems almost exclusively from assuming zero explantations over 15 years. This places extraordinary weight on an assumption derived from inadequate evidence, transforming a small-sample observation into the primary driver of cost-effectiveness. In the scenario analysis where the explantation rate is set to a more realistic proxy input derived from another electromagnetic active BCHI, as reported by Caversaccio et al. [2], 92% of the estimated QALY gain is lost, demonstrating the extreme sensitivity to this parameter.

Compounding this error, zero-event probabilities were treated as fixed in probabilistic sensitivity analysis, excluding the main driver of utility differences from this important uncertainty assessment. Clinically, this approach fails to acknowledge the real-world uncertainty surrounding long-term Sentio performance.

## 4. Unvalidated Device Longevity Assumptions

The model assumes Sentio patients undergo reimplantation after 15 years while Osia patients require reimplantation after 10 years. With only six months follow-up, projecting Sentio longevity to 15 years represents extrapolation far beyond any reasonable evidence base. No clinical evidence demonstrates that patients actually experience these differential reimplantation timelines in real-world practice. Of particular note, the assumption for Sentio is inconsistent with the instructions for use, which explicitly state: “The lifetime for the Sentio Ti Implant is ten years” [5].

Reimplantation decisions involve multiple factors, one of which is progressive hearing loss severity, and the decision to explant requires clinical assessment and surgeon recommendation. Notably, the fitting range of Sentio’s electromagnetic transducer is limited to 45 dB HL compared with 55 dB HL for Osia’s piezoelectric transducer. As presented by Breitholtz et al. [6], the wider fitting range of Osia suggests it may compensate for age-related progression of hearing loss longer than Sentio, contradicting the authors’ longevity assumptions.

These unsubstantiated device longevity assumptions critically impact the cost-effectiveness conclusions. The modelled cost differential favouring Sentio ($4353) from delayed reimplantation accounts for approximately 62% of the total cost difference between devices ($7050).

## 5. Implications for Clinical and HTA Decision-Making

Cost-effectiveness analyses can inform decision-making, but their value depends entirely on methodological rigour and appropriate uncertainty handling. In the Värendh et al. study [1], the estimated QALY gain derives entirely from differences in modelled complication rates based on disparate evidence quality, not from demonstrated superior outcomes. Moreover, the majority of long-term cost differences rely on unsubstantiated device longevity assumptions.

For clinicians and other decision-makers considering BCHI options, this analysis does not provide reliable comparative effectiveness evidence and the conclusion of dominant cost-effectiveness should be viewed with considerable scepticism. Decisions should prioritise demonstrated patient outcomes, audiological performance data, and patient quality of life rather than a focus on economic models built on weak comparative evidence.

Establishing dominance requires rigorous comparative data on both clinical outcomes and costs. The Värendh et al. study [1] falls short of this standard and should not influence clinical practice patterns or HTA decisions without substantial correction or additional evidence.

## Data Availability

No new data were created or analyzed in this study. Data sharing is not applicable to this article.
